# Different artificial neural networks for predicting burnout risk in Italian anesthesiologists

**DOI:** 10.1186/s44158-025-00255-w

**Published:** 2025-07-01

**Authors:** Marco Cascella, Alessandro Simonini, Sergio Coluccia, Elena Giovanna Bignami, Gilberto Fiore, Emiliano Petrucci, Alessandro Vergallo, Giacomo Sollecchia, Franco Marinangeli, Roberto Pedone, Alessandro Vittori

**Affiliations:** 1 Unit of Anesthesiology, Intensive Care Medicine, and Pain Medicine, Department of Medicine, Surgery, and Dentistry, University of Salerno, Baronissi, Salerno, 84082 Italy; 2 Pediatric Anesthesia and Intensive Care Unit, Salesi Children’s Hospital, 60121 Ancona, Italy; 3 Branch of Medical Statistics, Biometry and Epidemiology “G. A. Maccacaro”, Department of Clinical Sciences and Community Health, Dipartimento di Eccellenza 2023–2027, Università degli Studi di Milano, Milan, 20133 Italy; 4 Anesthesiology, Critical Care and Pain Medicine Division, Department of Medicine and Surgery, University of Parma, Parma, 43121 Italy; 5 Department of Anesthesia and Intensive Care, Hospital of Santa Croce Di Moncalieri, Turin, 10024 Italy; 6 Department of Anesthesia and Intensive Care Unit, San Salvatore Academic Hospitalof, L’Aquila, 67100 Italy; 7 Department of Anesthesia and Intensive Care, Spedali Civili di Brescia, 25121 Brescia, Italy; 8 Department of Anesthesiology, Intensive Care and Pain Treatment, University of L’Aquila, L’Aquila, 67100 Italy; 9 Department of Psychology, University of Campania Luigi Vanvitelli, Caserta, 8100 Italy; 10 Department of Anesthesia, Critical Care and Pain Medicine, ARCO, Ospedale Pediatrico Bambino Gesù IRCCS, Rome, 00165 Italy

**Keywords:** Burnout, Anesthesiologists, Mental health, Artificial intelligence, Artificial neural networks, Anesthesia, Critical care, Pain, Critical care

## Abstract

**Background:**

Burnout (BO) is a serious issue affecting professionals across various sectors, leading to adverse psychological and occupational consequences, even in anesthesiologists. Machine learning, particularly neural networks, can offer effective data-driven approaches to identifying BO risk more accurately. This study aims to develop and evaluate different artificial dense neural network (DNN)-based models to predict BO based on occupational, psychological, and behavioral factors.

**Methods:**

A dataset (300 Italian anesthesiologists) comprising workplace stressors, psychological well-being indicators, and demographic variables was used to train DNN models. Model performance was measured using standard evaluation metrics, including accuracy, precision, recall, and F1 score. Statistical tests were adopted to assess differences in prediction across the DNNs.

**Results:**

The best neural architecture achieved a predictive accuracy of 0.68, with key contributors to BO including workload, emotional exhaustion, job dissatisfaction, and lack of work-life balance. Despite substantial differences among the six implemented algorithms, no significant variation in prediction performance was observed.

**Conclusion:**

Psychological distress scores are significantly higher in the high-risk BO group, suggesting greater anxiety, depression, and overall distress in this category. While challenges remain, continued advancements in artificial intelligence and data science promise more effective and personalized mental health care solutions.

**Trial registration:**

Not applicable.

**Supplementary Information:**

The online version contains supplementary material available at 10.1186/s44158-025-00255-w.

## Background

Burnout (BO) is an occupational phenomenon, described by the World Health Organization as a “syndrome resulting from chronic workplace stress that has not been effectively managed” [1]. This syndrome manifests through three dimensions, encompassing “feelings of energy depletion or exhaustion (emotional exhaustion); increased mental distance from one’s job (depersonalization), or feelings of negativism or cynicism related to one's job; and reduced professional efficacy (personal accomplishment)” [2]. In healthcare professionals (HCPs), these manifestations can have significant implications for professional and personal well-being [3]. Significantly, BO demonstrates a prevalence that can escalate to 50% in specific categories facing an elevated risk of stress [4, 5]. Previous reports have specifically highlighted that approximately half of intensivists experience a high level of BO, with up to 59% of anesthesiologists displaying some features indicative of high-risk BO [6, 7].

Previously, we conducted an analysis of BO risk among anesthesiologists in Italy [8, 9]. The proposed questions addressed the work environment, the private sphere, and the relationship between work and family life. Descriptive analyses highlighted that one-third of the sample could be considered at low risk of BO, another third at medium risk, and the remaining third at high risk. The latter group showed classic symptoms of BO — emotional exhaustion, depersonalization, and lack of motivation — manifesting with significant impact on both work and personal life.

Stressors such as long working hours, conflicts with surgeons, and interpersonal disputes within the specialty were identified as particularly burdensome.

Accordingly, in a study by cardio-anesthesiologists, the findings revealed that 34%, 54%, and 66% of respondents fell into the categories of “high” or “moderate-high” risk for BO in terms of emotional exhaustion, depersonalization, and personal accomplishment, respectively [10].

Despite its well-characterized dimensions, BO remains a complex and multifactorial syndrome, shaped by individual, interpersonal, and systemic factors [11]. Its subjective manifestations, variability across individuals, and dynamic evolution over time pose significant challenges to early identification and risk stratification. Traditional assessment tools often rely on retrospective self-reporting and may fall short in capturing the nuanced interplay of variables contributing to BO.

In this complex scenario, artificial intelligence (AI) and machine learning (ML) provide promising avenues for addressing these challenges [12]. Advanced algorithms and statistical models offer powerful tools for predictive analytics, enabling the processing of large, heterogeneous datasets, the identification of hidden patterns, and the generation of personalized risk profiles. Therefore, AI-based technologies are particularly well-suited to model complex phenomena like BO, where interactions between multiple variables — workload, interpersonal dynamics, personal resilience, and institutional factors — must be considered simultaneously. Moreover, the adaptive nature of ML algorithms allows for continuous learning from new data, enhancing predictive accuracy over time and supporting dynamic, real-time risk monitoring [10, 13, 14].

Based on the collected data, we developed a predictive model by implementing AI approaches. The aim is to evaluate whether an AI-based predictive model can provide a proactive, data-driven approach to managing BO among anesthesiologists, with a focus on early detection, personalized support, and targeted interventions to enhance the well-being of this group of HCPs.

## Methods

The research was granted ethical approval by the Ethics Committee of L'Aquila and Teramo under protocol number 0024436/20 (Chairman Dr. Goffredo Del Rosso). All participants willingly volunteered after receiving a thorough explanation of the study, and written informed consent was obtained from all the subjects, and their involvement adhered to the “Ethical Principles of Psychologists and Code of Conduct.” All methods were performed in accordance with the ethical standards as laid down in the Declaration of Helsinki and its later amendments or comparable ethical standards.

This manuscript adheres to the applicable Strengthening the Reporting of Observational Studies in Epidemiology (STROBE) guidelines (www.strobe-statement.org).

### Study design and participants

This study was conducted using a dataset collected through a structured questionnaire administered in person to participants attending refresher courses at the AAROI-EMAC (Italian Association of Hospital Anesthesiologists, Pain Medicine Specialists, Critical Care, and Emergency Physicians) Simulation Center in February 2020. The sample consisted exclusively of physicians who were active members of AAROI-EMAC, an organization with approximately 10,000 registered members [9]. To ensure the reliability of the findings, only practicing physicians were included in the study. Individuals with a self-reported history of psychiatric disorders, substance-related conditions, or psychotropic medication use were excluded from participation. Before data collection, participants received a detailed explanation of the study’s objectives and voluntarily consented to participate.

The questionnaire comprised seven sections designed to assess multiple aspects related to BO and occupational well-being through sociodemographic questions (SG). The sections included demographic data (e.g., age, gender, education), turnover intent, personality traits, BO assessment, work engagement, work context, and job satisfaction (Appendix 1).

The final dataset was registered on Zenodo [15].

#### Trial registration

This is not applicable.

### Burnout measurement

The Maslach Burnout Inventory (MBI) was used to assess BO levels across three key dimensions including emotional exhaustion (EE), measured by nine items (e.g., “I feel emotionally drained from my work”); depersonalization (DP), assessed through five items reflecting detachment from work and colleagues (e.g., “I feel I treat some people as if they were impersonal objects”); and personal accomplishment (PA), evaluated through eight items related to work-related self-efficacy (e.g., “I feel I’m positively influencing other people’s lives through my work”) [16].

Each item was rated on a 7-point Likert scale (0 = *never* to 6 = *every day*), with BO risk categorized based on predefined score thresholds. The validated Italian version of the MBI was used, with internal consistency coefficients of *α* = 0.72 for the overall scale, *α* = 0.88 (EE), *α* = 0.75 (DP), and *α* = 0.76 (PA) for the subscales [17].

### Additional psychological measures

- Toronto Alexithymia Scale (TAS-20): A 20-item self-report measure that assesses alexithymia, with each item rated on a 5-point Likert scale [18, 19]
- Symptom Checklist-90-R (SCL-90-R): A 90-item self-report inventory that measures psychological symptom status [20, 21]
- Rosenberg Self-Esteem Scale (RSES): A list of 10 closed questions that measures global explicit self-esteem [22]
- State-Trait Anxiety Inventory-Trait version (STAI-T): A list of 20 statements that individuals rate based on their general feelings, such as calmness, confidence, or security, to measure a stable personality trait related to anxiety proneness [23]
- Beck’s Depression Inventory (BDI): A 21-item, self-report rating inventory that measures characteristic attitudes and symptoms of depression [24]

### Dataset preprocessing

The initial sample consisted of 300 Italian anesthesiologists. Individuals with missing data or reporting unspecified qualifications (i.e., “Other”) were excluded from the analysis. Continuous data was centered and standardized, while one-hot encoding was applied to categorical data, dropping the reference category.

### Artificial neural network

Artificial neural network (ANN) architectures were utilized to predict the target feature (i.e., the status of BO) [25]. A dense neural network (DNN) architecture was used. It is a rich and complex set of nested functions aiming to provide the lowest error overcoming between prediction and actual outcome values from the set of available features. Such functions are also called neurons or nodes and are distributed in cascade layers; each layer receives the input values from the preceding neurons and calculates their value according to some coefficients (weights), and so on, ending with the output layer (even a single neuron) which generates a theoretical output value. The backpropagation method consists of letting the output enter back and forth cycles, leading the net to optimize the weights, which regularize the force of information propagation by any single neuron. The learning phase of the model flows through the minimization of a loss function given the information, e.g., the features, from the training set, which enter the cycle multiple times. One epoch is the frequency of training the neural network with all the training data for one cycle. This method leads to a more powerful efficiency in terms of output prediction but also to be heavier in terms of overhead.

The DNN models were chosen according to the size of the sample, obtaining a triangular-like shape. The target feature was treated as continuous (0 = moderate risk of BO, 1 = high risk of BO), and the mean square error (MSE) was used both as a metric and loss function. Thus, the risk was considered as the actual probability of incurring a high risk of BO. Data was balanced to provide more boost to the models (Table 1).

**Table 1:** Descriptive and univariable analysis (*n* = 269)

Characteristic	Descriptive analyses	Univariable by burnout risk		
	*N* = 269^1^	Moderate, *N* = 139^1^	High, *N* = 130^1^	*p*-value
Gender				> 0.999^2^
* Male*	91 (33.8%)	47 (33.8%)	44 (33.8%)	
* Female*	178 (66.2%)	92 (66.2%)	86 (66.2%)	
Age				0.910^3^
* Mean (SD)*	44.6 (9.3)	44.9 (9.7)	44.4 (9)	
* Median (IQR)*	43 (37, 52)	44 (38, 52)	43 (37, 50)	
Degree				0.933^2^
* Postgraduate course and* *postgraduate specialization*	233 (86.6%)	120 (86.3%)	113 (86.9%)	
* Advanced training*	21 (7.8%)	12 (8.6%)	9 (6.9%)	
* Doctorate*	15 (5.6%)	7 (5%)	8 (6.2%)	
Not ICU — not anesthesia				0.071^2^
* No*	201 (74.7%)	112 (80.6%)	89 (68.5%)	
* Yes*	68 (25.3%)	27 (19.4%)	41 (31.5%)	
SG1				0.677^2^
* No*	115 (42.8%)	57 (41.0%)	58 (44.6%)	
* Yes*	154 (57.2%)	82 (59%)	72 (55.4%)	
SG3				0.267^3^
* Mean (SD)*	6.7 (1.5)	6.7 (1.7)	6.7 (1.2)	
* Median (IQR)*	6 (6, 7)	6 (6, 7)	6 (6, 7)	
SG6				0.001^2^
* No*	40 (14.9%)	10 (7.2%)	30 (23.1%)	
* Yes*	229 (85.1%)	129 (92.8%)	100 (76.9%)	
SG7				> 0.999^2^
* No*	33 (12.3%)	17 (12.2%)	16 (12.3%)	
* Yes*	236 (87.7%)	122 (87.8%)	114 (87.7%)	
SG13				0.075^2^
* North*	143 (53.2%)	64 (46.0%)	79 (60.8%)	
* Middle Italy*	71 (26.4%)	39 (28.1%)	32 (24.6%)	
* South and Islands*	55 (20.4%)	36 (25.9%)	19 (14.6%)	
SG15				> 0.999^2^
* No*	215 (79.9%)	111 (79.9%)	104 (80%)	
* Yes*	54 (20.1%)	28 (20.1%)	26 (20%)	
SG16				0.398^2^
* Freelancer*	24 (8.9%)	16 (11.5%)	8 (6.2%)	
* Precarious worker*	31 (11.5%)	15 (10.8%)	16 (12.3%)	
* Stable worker*	214 (79.6%)	108 (77.7%)	106 (81.5%)	
SG17				0.267^2^
* No*	246 (91.4%)	124 (89.2%)	122 (93.8%)	
* Yes*	23 (8.6%)	15 (10.8%)	8 (6.2%)	
SG24				0.092^2^
* No*	209 (77.7%)	101 (72.7%)	108 (83.1%)	
* Yes*	60 (22.3%)	38 (27.3%)	22 (16.9%)	
SG25				> 0.999^4^
* No*	260 (96.7%)	134 (96.4%)	126 (96.9%)	
* Yes*	9 (3.3%)	5 (3.6%)	4 (3.1%)	
SG27				0.008^2^
* No*	93 (34.6%)	36 (25.9%)	57 (43.8%)	
* Yes*	176 (65.4%)	103 (74.1%)	73 (56.2%)	
SG28				0.291^2^
* No*	128 (47.6%)	61 (43.9%)	67 (51.5%)	
* Yes*	141 (52.4%)	78 (56.1%)	63 (48.5%)	
SG29				0.107^2^
* No*	118 (43.9%)	53 (38.1%)	65 (50%)	
* Yes*	151 (56.1%)	86 (61.9%)	65 (50%)	
SG30				0.092^2^
* No*	152 (56.5%)	87 (62.6%)	65 (50%)	
* Yes*	117 (43.5%)	52 (37.4%)	65 (50%)	
SG31				0.048^2^
* No*	126 (46.8%)	55 (39.6%)	71 (54.6%)	
* Yes*	143 (53.2%)	84 (60.4%)	59 (45.4%)	
SG32				0.198^2^
* No*	226 (84%)	112 (80.6%)	114 (87.7%)	
* Yes*	43 (16%)	27 (19.4%)	16 (12.3%)	
SG34				0.283^2^
* No*	248 (92.2%)	131 (94.2%)	117 (90%)	
* Yes*	21 (7.8%)	8 (5.8%)	13 (10%)	
SG35				0.185^2^
* No*	32 (11.9%)	21 (15.1%)	11 (8.5%)	
* Yes*	237 (88.1%)	118 (84.9%)	119 (91.5%)	
SG46				0.452^2^
* No*	32 (11.9%)	19 (13.7%)	13 (10%)	
* Yes*	237 (88.1%)	120 (86.3%)	117 (90%)	
SG48				0.198^2^
* No*	184 (68.4%)	89 (64%)	95 (73.1%)	
* Yes*	85 (31.6%)	50 (36%)	35 (26.9%)	
SG49				0.075^2^
* No*	223 (82.9%)	122 (87.8%)	101 (77.7%)	
* Yes*	46 (17.1%)	17 (12.2%)	29 (22.3%)	
SG50				0.267^2^
* No*	218 (81.0%)	117 (84.2%)	101 (77.7%)	
* Yes*	51 (19%)	22 (15.8%)	29 (22.3%)	
SG51				0.008^2^
* No*	210 (78.1%)	119 (85.6%)	91 (70%)	
* Yes*	59 (21.9%)	20 (14.4%)	39 (30%)	
STAIT				< 0.001^3^
* Mean (SD)*	39.4 (8.6)	36.1 (7.1)	43.0 (8.6)	
* Median (IQR)*	38 (34, 44)	36 (31, 40)	42 (37, 48)	
SCL90				< 0.001^3^
* Mean (SD)*	0.6 (0.4)	0.4 (0.2)	0.7 (0.5)	
* Median (IQR)*	0.5 (0.3, 0.7)	0.4 (0.2, 0.6)	0.6 (0.3, 0.9)	
BDItot				< 0.001^3^
* Mean (SD)*	6.8 (6.1)	5.1 (5.2)	8.8 (6.4)	
* Median (IQR)*	6.0 (2, 10)	4.0 (1.5, 7)	8 (4, 12)	
TASTOTr				< 0.001^3^
* Mean (SD)*	42.9 (8.9)	40.8 (8)	45.2 (9.2)	
* Median (IQR)*	42 (37, 48)	40 (35.5, 46)	44 (39, 50.8)	
RSEGS				< 0.001^3^
* Mean (SD)*	22.9 (4.9)	24.2 (4.6)	21.6 (5)	
* Median (IQR)*	24 (20, 27)	25 (22, 27)	22 (18, 25)	

^1^*n* (%); *p*-values were corrected adopting the Benjamini-Hochberg “false discovery rate” correction

^2^Pearson’s chi-squared test

^3^Wilcoxon rank-sum test

^4^Fisher’s exact test

The sample was split into a train and test set in an 80/20 ratio: DNNs were on 216 observations (80% of the sample) and tested on the remaining subsample (*n* = 53). A k-fold cross-validation technique was assessed to detect the optimal number of epochs for training up to 200, with *k* = 4. According to this method, the training set is split into k parts. Each part was subsequently split into internal train (70%) and validation (30%) subsets and fed to the model to fit it across an aforethought set of hyperparameters, in our case the number of epochs. The optimal number of epochs was detected as the value corresponding to the lower value (e.g., the “argmin”) of the average of the MSEs across the four model fittings. The early stopping parameter, fixed to the squared root of the number of epochs (*n* = 14), was set to prevent null-learning or overfitting during training.

Performance measures were presented as main statistics reading binary data output, and the best cutoff for maximization of the AUC-ROC (area under the receiver operating characteristic curve) was evaluated. Other performances were calculated by assessing the main confusion matrix statistics.

Various sizes of DNNs were developed (see Table 2). A tanh activation function was set for the first hidden layer and then a ReLU (rectified linear unit) one; an Adam optimizer was adopted, with a learning rate and epsilon fixed as 10^−3^.

**Table 2:** ANN structures and performances

Model	No. of hidden layers	Number of neurons by layer	No. of parameters	No. of epochs	Optimal cutoff	AUC-ROC on train set	AUC-ROC on test set
1	4	24, 16, 10, 6	1507	166	0.44	0.88	0.60
2	3	16, 10, 4	795	156	0.40	0.93	0.58
3	3	10, 6, 3	451	40	0.32	0.70	0.62
4	2	24, 10	1125	66	0.46	0.92	0.66
5	2	16, 6	685	111	0.49	0.83	0.68
6	1	16	593	165	0.47	0.89	0.60

### Models’ assessment

Several metrics were used to dissect the models’ predictive power, generalization ability, and reliability.

- Accuracy is the proportion of correctly classified cases out of the total cases. It indicates overall model performance. The no-information rate (NIR) represents the accuracy that would be achieved by randomly guessing the outcome, e.g., predicting the most frequent class in the dataset. The parameter serves as a baseline to compare the model’s performance. A significantly higher model accuracy compared to NIR suggests the model is effective.
- Sensitivity (recall) is the ability to correctly identify high-risk cases (true positives). A higher value indicates fewer false negatives.
- Specificity is the ability to correctly identify medium–low risk cases (true negatives). A higher value indicates fewer false positives.
- The F1 score is the harmonic mean of precision and recall, balancing false positives and false negatives. It is particularly useful when classes are imbalanced.
- Kappa score represents a measure of agreement between the model’s predictions and actual classifications, adjusting for chance agreement. Higher values indicate better reliability.
- The AUC-ROC measures the model’s ability to distinguish between classes. Higher values indicate better classification performance.

### Statistics

Descriptive statistical analyses were conducted using Pearson’s chi-squared test or Fisher’s exact test for categorical variables and the Wilcoxon rank-sum test for continuous variables [26–28]. To account for multiple comparisons, *p*-values were adjusted using the Benjamini–Hochberg false discovery rate correction [29]. All the performance statistics described in the paragraph above were drawn from both training and test sets, showed and commented. Delong’s test for AUC comparisons was performed across all the couples to investigate possible differences between models [30].

All the analyses and plots were realized in R (v. 4.3.2); specifically, the Python environment (v. 3.8.0) was set to use TensorFlow and Keras modules in the R environment, and different packages (caret, purrr, ROCR, pROC, pRROC) were implemented for graphs and performance calculations [31].

## Results

The descriptive and univariable analysis concerned BO risk among 269 participants (269 questionnaires completed in every part out of 300), divided into moderate (*n* = 139) and high-risk (*n* = 130) groups. The gender distribution was identical between the groups, with 33.8% males and 66.2% females (*p* > 0.9992). The average age was similar across groups, with a mean of 44.6 years (*SD* = 9.3) and no significant difference (*p* = 0.9103). Regarding education, most participants, in addition to specialization in anesthesia and intensive care, hold a postgraduate course or postgraduate specialization (86.6%), followed by those with advanced training (7.8%) and a doctorate (5.6%), with no significant variations between BO risk groups (*p* = 0.9332).

When considering work-related factors, a higher proportion of individuals in the high-risk group declared to work in services compared to the moderate-risk group (31.5% vs. 19.4%, *p* = 0.0712). Most participants had a permanent contract (79.6%), and no significant differences were observed between the groups (*p* = 0.3982).

The geographical distribution demonstrated that individuals from Northern Italy are more frequent in the high-risk group (60.8% vs. 46.0%, *p* = 0.0752), while those from the South and Islands are less represented in the high-risk group compared to the moderate-risk group (14.6% vs. 25.9%).

Several psychological variables showed significant differences between BO risk groups. Specifically, sociodemographic groups (SG) SG6 (*p* = 0.0012), SG27 (*p* = 0.0082), SG51 (*p* = 0.0082), and SG31 (*p* = 0.0482) indicate notable disparities. Moreover, psychological distress scores, including STAIT, SCL90, BDItot, TAStotr, and RSEGS, were significantly higher in the high-risk BO group (*p* < 0.0013), suggesting greater anxiety, depression, and overall distress in this category. Descriptive characteristics are summarized in Table 1.

### Artificial neural network

The obtained DNNs varied in the number of hidden layers, neurons per layer, total parameters, and performances. The computational time varied from 45.9 s for Model 3 to 2.9 min to fit Model 6 and Model 1. Model 5 recorded the third lowest time (2.6 min). Complete results are consultable in Table 2. The number of epochs varied across models, with Models 1 and 6 with the highest values (166, 165, respectively) and Models 3 (40) and 4 (66) achieving lower values. Model 1 was built with the highest number of hidden layers (*n* = 4), reached 1507 parameters, and achieved an AUC-ROC of 0.88 on the training set and 0.60 on the test set. Model 2, with 3 hidden layers and 795 parameters, demonstrated the highest AUC-ROC on the training set (0.93) but a lower test set (0.58). Model 3, which was more compact with 3 layers and only 451 parameters, reached an AUC-ROC of 0.70 (train) and 0.62 (test). Model 4, with 2 layers and 1125 parameters, exhibited the best performance (*AUC-ROC* = 0.92) on the training set but reached an AUC of 0.66 on the test set. Model 5, with two hidden layers and 685 parameters, performed best on the test set (*AUC-ROC* = 0.68), indicating better generalization. Finally, Model 6, the simplest structure with a single hidden layer, achieved an AUC-ROC of 0.89 on the training set and 0.60 on the test set.

These results suggest that while deeper models with more parameters may have excelled in training, they did not necessarily generalize better, as seen in the nominally superior test performance of the shallower Model 5.

**Fig. 1:**
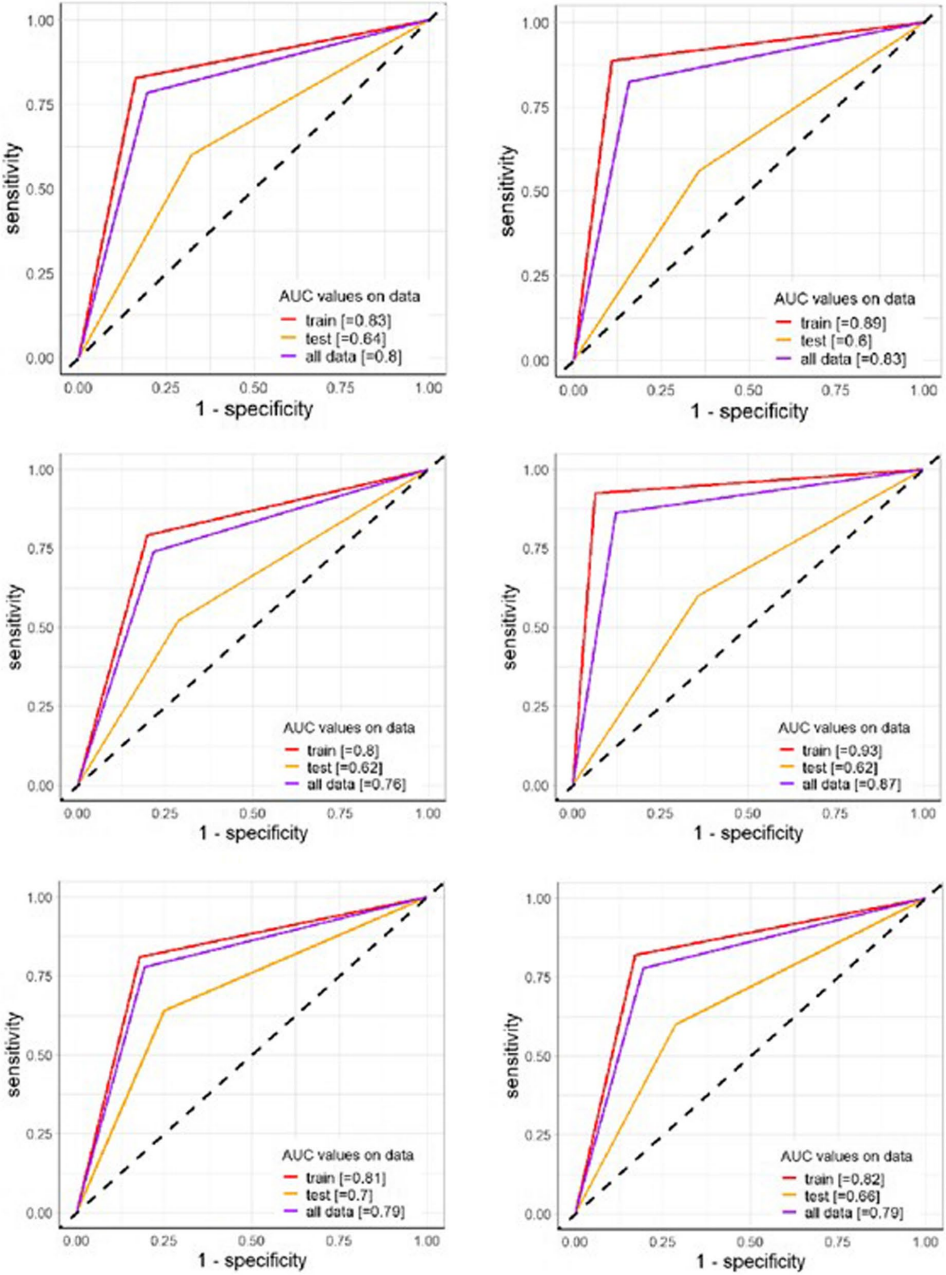
AUC-ROCs graphs by algorithm. Figures refer to models from 1 (top left) to 6 (bottom right). AUC-ROC statistics range between 0.66 and 0.8 on the train set and from 0.60 to 0.70 on the test set. The best performance on the test set was reached by models 5 and 6 (0.70 and 0.66, respectively), which are reconnectable to the lighter models

Delong’s test for difference in ROC curves revealed no statistical significance among ROCs comparison (*p* > 0.10 for all the tests). Figure 1 shows the overall performance of the models for training and testing.

Concerning the parameters of the best model (Model 5), in the training set, it correctly classified 81 medium–low risk cases and 87 high-risk cases while misclassifying 18 cases in each category. In the test set, the model correctly predicted 21 medium–low risk and 15 high-risk cases while misclassifying 7 medium–low risk cases as high risk and 10 high-risk cases as medium–low risk. Moreover, the model achieved an accuracy of 0.83 (95% *CI*: 0.78–0.88) on the training set and 0.68 (95% *CI*: 0.54–0.80) on the test set. Sensitivity and specificity were > 0.80 for training and 0.68 for testing, respectively. NIR was 0.51 for the training set and 0.58 for the test set. This means that if we always predicted the most common category (either “medium–low risk” or “high risk”), we would be correct 51% of the time in the training set and 58% of the time in the test set. The *p*-value (< 0.01 for training and 0.10 for testing) tests whether the model’s accuracy is significantly better than the NIR, with a weaker indication even for the test set. The F1 score was 0.83 for training and 0.64 for testing, while the kappa score, which measures agreement beyond chance, was 0.67 for training and 0.35 for testing. These results indicate that while Model 5 performed well on training data, its generalization to unseen test data was slightly lower, as reflected in reduced accuracy, F1 score, and kappa score (Table 3).

**Table 3:** Global performance metrics of the best ANN model (model 5) on the training (*n* = 216) and test (*n* = 53) sets

		Train data (*n* = 216)			Test data (*n* = 53)	
		*Prediction*			Test data (*n* = 53)	
		Medium-low risk	High risk		Medium-low risk	High risk
Observed	Medium-low risk	93	18	Medium-low risk	21	7
	High risk	18	87	High risk	10	15
Accuracy (95% CI)		0.83 (0.78, 0.88)			0.68 (0.54, 0.80)	
P [Acc≠NIR]		*NIR* = 0.51< 0.01			*NIR* = 0.580.10	
Sensitivity		0.83			0.68	
Specificity		0.84			0.68	
F1 score		0.83			0.64	
Kappa score		0.67			0.35	

## Discussion

Despite limitations, the results of this analysis demonstrate that AI-based applications can provide valuable insights for assessing BO levels in a selected category of healthcare workers [32]. Specifically, AI strategies can be effectively employed to identify individuals at risk of the syndrome [33]. The aim is to facilitate early interventions and personalized support strategies [32].

One of the key findings of this study is the high predictive accuracy achieved by the ANN model. After careful performance assessment, the best model (i.e., Model 5) demonstrated a strong ability to differentiate between different BO levels based on input variables. Therefore, the selected features during preprocessing played a significant role in determining BO risk. Notably, these results align with previous research indicating that ML can successfully analyze psychological and physiological data to predict mental health conditions [34–36].

Furthermore, the use of neural networks allows for the identification of complex, nonlinear relationships between variables, which traditional statistical methods might not fully capture. This highlights the advantage of using AI-based techniques in psychological assessments, where multiple interacting factors contribute to an individual’s well-being [37]. In addition, natural language processing (NLP) and large language models (LLMs) can play a key role by enabling the analysis of unstructured textual data such as clinical notes, open-ended survey responses, or patient narratives. This combined approach can provide deeper insights into psychological states, enhance the accuracy and personalization of assessments, and guide preventive strategies. For example, the integration of gamification processes and other educational modalities presents promising prospects for increasing user engagement, reducing response fatigue, and facilitating the continuous monitoring of psychological well-being through interactive, user-friendly platforms [38].

Since BO is a concrete professional risk, it is necessary to direct research along two lines: developing policies capable of correcting or eliminating the environmental factors capable of determining BO and the ability to predict BO early. To better identify the most effective health policies for reducing or eliminating the risk of BO, we chose to integrate the practical expertise of AAROI-EMAC—a professional association with in-depth knowledge of the working conditions of Italian anesthesiologists — with the scientific contribution of a psychologist expert in the field, who developed a questionnaire designed to complement the MBI.

In the first publication resulting from the study, some interesting data emerged, surprising in some respects, which should make us reflect precisely because they were collected before the Covid-19 pandemic, therefore in a normal situation. Health policies are certainly the most powerful weapon to combat BO because they can undermine the distortions present in the working system such as organizational, economic, and career progression aspects. However, health policies also have limitations when addressing BO. As previously noted, BO is a contextualized occupational risk, and while health policies may be specific, they are often framed within national or regional contexts — such as in Italy — potentially limiting their adaptability to diverse local realities.

### Limitations

The first limitation is that the dataset size may impact the generalizability of the findings. For example, the low number of epochs required to reach convergence is indicative of a sample that is too small. Therefore, although the model performed well within the study’s scope, its applicability to broader populations remains to be validated with larger and more diverse samples. Secondly, while the best AI architecture provided significant predictive power, the interpretability of the model remains a challenge. Deep learning models often function as “black boxes,” making it difficult to extract clear causal relationships between variables. Future research should explore methods to enhance explainability, such as SHAP values or feature importance analysis [10].

BO represents a recognized occupational risk for all anesthesiologists worldwide. However, the literature agrees in stating that BO must be contextualized not only to the type of work but also to the national context in which it is carried out. Results obtained in a certain country may not be found in another application. Accordingly, we targeted a representative sample of professionals from across all regions of Italy.

## Conclusions

The implementation of AI-driven predictive models in organizational settings could revolutionize BO prevention strategies. Employers and mental health professionals could use these models to monitor employees’ well-being continuously and intervene proactively when signs of burnout emerge. However, ethical considerations, such as data privacy and the responsible use of AI in mental health, must be carefully addressed. Future studies should aim to refine the ANN architecture, test alternative ML models, and integrate multimodal data sources to enhance predictive performance. Notably, natural language models could offer interesting perspectives. Additionally, longitudinal studies would be beneficial to assess how well AI models predict BO over time and whether early detection leads to effective interventions.

## Supplementary Information

Supplementary Material 1: Appendix 1. Socio-demographic questions.

## Data Availability

The datasets used and/or analysed during the current study are available from the corresponding author on reasonable request.

## Abbreviations

AAROI-EMAC: Italian Association of Hospital Anesthesiologists, Pain Medicine Specialists, Critical Care, and Emergency Physicians
AI: Artificial intelligence
ANN: Artificial neural network
AUC: Area under the curve
AUC-ROC: Area under the receiver operating characteristic curve
BDI: Beck’s Depression Inventory
BO: Burnout
CI: Confidence interval
DNN: Dense neural network
DP: Depersonalization
EE: Emotional exhaustion
HCPs: Healthcare professionals HCPs
ICU: Intensive care unit
IQR: Interquartile range
MBI: Maslach Burnout Inventory
ML: Machine learning
MSE: Mean square error
P_NIR: No-information rate
PA: Personal accomplishment
ReLU: Rectified linear unit
ROC: Receiver operating characteristic
RSEGS: Rosenberg Self-Esteem Scale global score
RSES: Rosenberg Self-Esteem Scale
SCL90GSI: SCL90 Global Severity Index
SCL-90-R: Symptom Checklist-90-R
SD: Standard deviation
SG: Sociodemographic question
SHAP: SHapley Additive exPlanations
STAI-T: State-Trait Anxiety Inventory-Trait version
TAS-20: Toronto Alexithymia Scale
TASTOTr: Toronto Alexithymia Scale total score
